# Alternating Electric Field-Based Static Gesture-Recognition Technology

**DOI:** 10.3390/s19102375

**Published:** 2019-05-23

**Authors:** Haoyu Wei, Pengfei Li, Kai Tang, Wei Wang, Xi Chen

**Affiliations:** State Key Laboratory of Mechatronics Engineering and Control, Beijing Institute of Technology, Beijing 100081, China; 2120160258@bit.edu.cn (H.W.); tangkai01@bit.edu.cn (K.T.); wywei@bit.edu.cn (W.W.); chenxi@bit.edu.cn (X.C.)

**Keywords:** human–computer interaction, gesture recognition, electric-field detection

## Abstract

Currently, gesture recognition based on electric-field detection technology has received extensive attention, which is mostly used to recognize the position and the movement of the hand, and rarely used for identification of specific gestures. A non-contact gesture-recognition technology based on the alternating electric-field detection scheme is proposed, which can recognize static gestures in different states and dynamic gestures. The influence of the hand on the detection system is analyzed from the principle of electric-field detection. A simulation model of the system is established to investigate the charge density on the hand surface and the potential change of the sensing electrodes. According to the simulation results, the system structure is improved, and the signal-processing circuit is designed to collect the signal of sensing electrodes. By collecting a large amount of data from different operators, the tree-model recognition algorithm is designed and a gesture-recognition experiment is implemented. The results show that the gesture-recognition correct rate is over 90%. With advantages of high response speed, low cost, small volume, and immunity to the surrounding environment, the system could be assembled on a robot that communicates with operators.

## 1. Introduction

User-centered, harmonious and natural human–computer interaction technology has gradually become a research hotspot [1,2]. This kind of communication requires the input device to meet the user’s behavior as far as possible. Traditional mechanical input devices find it difficult to realize input in 3D and with high degree of freedom, while gestures have strong information transmission capabilities that can express user’s intentions vividly and intuitively. Therefore, human–computer interaction technology based on gesture recognition has important research value.

A static gesture refers to a hand shape or posture that is stationary, while a dynamic gesture can be viewed as continuous static gestures on the time axis. In a virtual reality system, operators can interact with a virtual system through static gestures. Meanwhile, in a gesture-control system, operators can use static gestures to control the activity of the robot. The research on gesture interaction is very extensive, but because of the ambiguity and time-space difference of gestures, gesture recognition has been difficult to achieve at high resolution in some fields. Current main gesture-detection technologies include wearable device-based technology and computer vision information-based technology. A gesture-recognition system based on the wearable devices can accurately reflect the movement intention of the human body, but measurement equipment needs to be attach to the human body, which has a negative impact on the user experience. The application of gesture-recognition systems based on visual information is very promising [3,4], but there are some limitations in some respects. When the lighting conditions are insufficient, the background is complicated, or the hand is in front of the body, it is extremely difficult to completely separate and extract the human hand from the background and the body [2]. At the same time, in the process of tracking gestures, the detection system tends to have a large amount of manipulation due to the feature quantity of the human gestures and the number of gesture samples, and thus delays. Therefore, coherence and real-time capability in the interaction process needs to be improved.

With the rapid development of computer and digital signal-processing technology, the detection performance of three-dimensional optical systems based on active illumination has been greatly improved in recent years. Typically, RGB-D sensors use infrared imaging technology to acquire depth information of the human body based on the triangulation principle [5,6]. In 2013, a somatosensory controller that could accurately capture hand movements came out, called leap motion, which has the same principle as RGB-D sensors, with a recognition accuracy of up to 0.01 mm and a maximum acquisition frame rate of 200 frames per second [7,8]. In addition, sensors using the Time of Flight detection principle are also gradually applied to close-range measurements [9]. At present, active stereo vision technology is widely used in the field of robots. The detection system usually uses structured light imaging technology to obtain the depth information of the target by analyzing the phase or code [10]. However, such optical systems still face similar problems as traditional two-dimensional optical sensors. complex and varied outdoor environments can affect the normal operation of the system.

In recent years, electric-field detection technology has been gradually developed and extensively concerned [11,12,13], especially for the detection of the human body [14,15,16,17,18]. The key parts of the electric-field detection system are the emitting electrode and the sensing electrode. The emitting electrode radiates an electric field to the space under the action of an alternating excitation source, and the sensing electrode generates induced charge in the electric field. When a hand enters the electric field, the quantity of charge on the hand is redistributed, disturbing the original electric field. It causes a change in the quantity of charge on the sensing electrode, thereby generating induced current. The posture and position of the hand can be determined according to the current changes on different sensing electrodes. Since 1995, Smith’s team from MIT has developed a human–computer interaction system for positioning human hands in 3D space. He has equalized the human hand to three collinear spheres with the same spacing, which can determine whether the hand is in flat, raised or drooping state [19,20,21,22]. In 2007, Jaeseon Lee et al. developed an electric field-based gesture-recognition panel, where static gestures are recognized by detecting changes in the capacitance between orthogonally arranged emitting and sensing conductors on the panel [23], but without further research on the differences in users’ gestures. Differences will lead to users repeating the same gestures, where the hand is possibly sagging, up, sidespinning, or offset, so the panel capacitance will also change differently, impacting gesture recognition results. In 2013, the Microchip Corporation of the United States designed a three-dimensional gesture recognition system to track the center of the hand charge [24], to obtain the position and trajectory information of the hand movement, but the system cannot identify the static gestures. In 2016, Gurashish Singh et al. also built a capacitor array system which could measure the movement of the hand [25].

In this paper, near-field sensing technology based on alternating electric field is adopted to study static gestures in different states. A new detection system and recognition algorithm are designed, realizing high accuracy of gesture recognition. In addition, the electric-field detection system also has a high recognition rate for dynamic gestures composed of static gestures. The detection system not only has high stability, not easily affected by the surrounding environment, but also has a high response speed to track changes in gestures in real time. Besides, the system cost is low, and the power consumption is small. Therefore, it can not only be used for gesture control of a social robot, but also can control the actions of people and the change of the scenes in virtual reality, with broad application prospects.

## 2. Method

### 2.1. Alternating Electric-Field Detection Principle

The electric-field detecting system consists mainly of an emitting portion and a sensing portion. The emitting portion includes an excitation source and an emitting electrode. The excitation source applies a sinusoidal alternating voltage to the emitter electrode, and the quantity of charge on the electrode changes with time, thereby forming an alternating electric field in space. Normally, the frequency f of the alternating voltage is between 10 and 150 kHz. It is chosen outside the frequency range of the radio to avoid being affected by radio signals. The electromagnetic wave wavelength λ=c/f generated by the charge on the electrode exceeds 2 km, and the detection distance of the system does not exceed 1 m. The emitting electrode is centimeter-sized, much smaller than the electromagnetic wavelength λ. Therefore, in the detection range of the system, the time-varying electromagnetic field can be regarded as quasi-static electrical near field at each moment. The electrostatic field equation can be used to solve the distribution of the electric field and potential in space. To improve the dynamic response characteristics of the system, the system carrier operates at a frequency between 40 and 100 kHz, and the performance of the system with a carrier frequency of 40 kHz is tested. 

The sensing portion includes sensing electrodes and signal-processing circuit. The sensing electrode generates induced current under the alternating electric field. The current is coupled to the circuit in the form of a voltage through a snubber capacitor. The signal-processing circuit amplifies and filters the input voltage, and then transmits it to the host computer through the data acquisition system. When the hand enters the detection area of the system, the charge on the hand is redistributed under the action of the electric field, causing disturbance to the original electric field. The distortion of the electric field is fed back to the signal receiving end of the detector, from which the system obtains the hand information and analyzes its position and posture.

**Figure 1:**
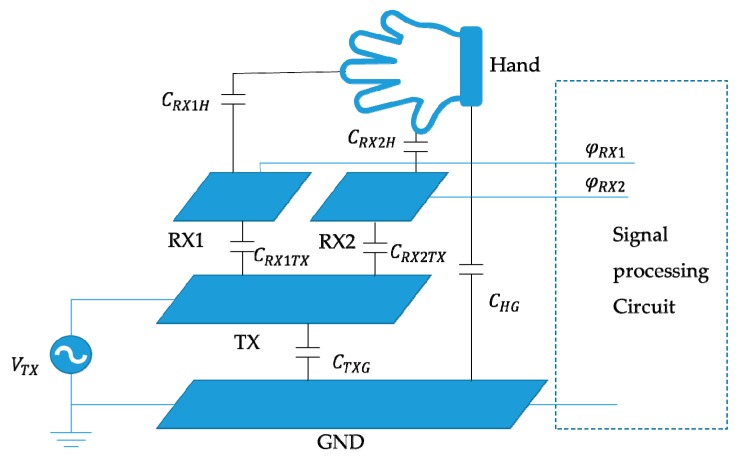
The equivalent circuit model of the electric-field detection system.

The equivalent circuit model of the electric-field detection system is shown in Figure 1. *RX1* and *RX2* are the sensing electrode, and the remaining electrodes are omitted from the model. *TX* is the emitting electrode. *GND* is the system ground. *V_TX_* is the excitation source. The capacitance between the emitting electrode and the system ground is *C_TXG_*, the capacitance between the sensing electrodes and the emitting electrode are *C_RX1TX_* and *C_RX2TX_*, respectively. Since the sensing electrodes are in the same plane, the capacitance between them is negligible. After the hand enters the electric field, the capacitance formed with the sensing electrodes are *C_RX1H_* and *C_RX2H_*, and the capacitance formed with the system ground is *C_HG_*. The change in the position of the hand causes a change in the capacitance between the hand and the sensing electrodes, and the induced charge *Q_RX_* of the electrode changes accordingly, thereby causing a change in the voltages *φ_RX1_* and *φ_RX2_* on the sensing electrodes. The detection system recognizes the gesture based on the difference in voltage changes of the electrodes.

When the hand does not enter the detection area, the charge density *σ_TX_* can be calculated according to the potential *V_TX_* of the emitting electrode, and the electric field intensity ***E**_TXRX0_* near the sensing electrode can be known, thus the induced voltage *φ_TXRX0_* can be expressed by the equation below.

(1) $$\varphi_{TXRX0}=\int_{RX}^{\infty }E_{TXRX0}\cdot dl$$

When the hand enters the electric field, the electric-field intensity *E_TXH_* near the hand can be known, according to Gauss’s law, the quantity of induced charge formed on the hand is *Q_h_*, which can be expressed as below.

(2) $$Q_{h}=\int_{S}\varepsilon n\cdot E_{TXH}dS$$

Since the curvature changes greatly on the hand, the distribution of charge is extremely uneven, so we divide it into several curved surfaces with different charge densities, with area of *S_i_*, charge density of *σ_i_*, and the distance from the sensing electrode of *R_i_* (*i* = 1, 2, …, n). The induced charge on the hand causes change to the spatial electric field, and the electric-field intensity ***E****_RX_* on the sensing electrode is deduced from above equation.

(3) $$E_{RX}=E_{TXRX0}+\frac{1}{4\pi \varepsilon }\sum_{i=1}^{n}\frac{\sigma_{i}S_{i}}{R_{i}^{2}}e_{R}$$

The sensing electrode potential *φ_RX_* can be calculated by integrating ***E****_RX_*. Calculate the change ∆*φ* of the potential of different sensing electrodes after the hand enters the electric field as follows.

(4) $$\Delta \varphi =\varphi_{RX}-\varphi_{TXRX0}$$

Because the shape of the human hand is irregular and cannot be solved analytically, the variation of the potential on the sensing electrode under different gestures is analyzed by simulation.

### 2.2. Design and Implementation of Electric-Field Detection System

The electric-field detection system is mainly composed of four parts, namely the sensing electrode, the insulating material, the emitting electrode, and the system ground. According to the function of each part, a reasonable system structure is designed, in which the system ground must be placed at the bottom of the model to shield the interference source below, ensuring that the sensing electrode is only affected by the emitting electrode and the hand above. The insulating material is above the system ground to isolate the system ground from other electrodes. The emitting electrode and the sensing electrode are above the insulating layer, which can be placed in the same layer or separately on the upper and lower layers. When the electrodes are in the same layer, the electric field formed by the emitting electrode in the space has less effect on the sensing electrode. The initial potential value of the sensing electrode is low. After the hand enters the detection range, the change in the potential of the sensing electrode will be small. By placing the sensing electrode above the emitting electrode, the change in the potential of the sensing electrode is significantly improved. Therefore, the hierarchical distribution of each part of the detection system is the sensing electrode layer, the insulating layer, the emitting electrode layer, the insulating layer, and the system ground from the top to the bottom, with a total of five layers.

**Figure 2:**
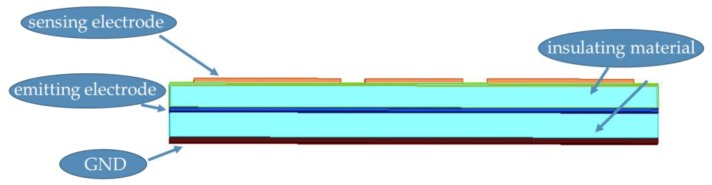
Electric-field detection system structure.

To improve the sensitivity of the system, it is necessary reasonably design the thickness of the insulating layer, increase the coupling degree between the hand and the sensing electrode, and reduce the coupling degree between the emitting electrode and the sensing electrode. When the system detection panel area is close to that of the hand, the area of the sensing electrode should be increased as much as possible to increase the capacitance *C_RX1H_* and *C_RX2H_* between the hand and the sensing electrode. Under the premise of meeting the portability requirements of the system, the distance between the sensing electrode and the emitting electrode is appropriately increased to reduce the capacitances *C_RX1TX_* and *C_RX2TX_* between them. In addition, the system ground and the emitting electrode should maintain a low degree of coupling, so as to reduce the effect of the system ground on the sensing electrode in the one hand, and to improve the driving capability of the excitation source on the other hand. Based on above analysis, the distance between the sensing electrode and the emitting electrode is determined to be 10 mm, and the distance between the emitting electrode and the ground is also 10 mm. On this basis, the model of the detection system is shown in Figure 2.

**Figure 3:**
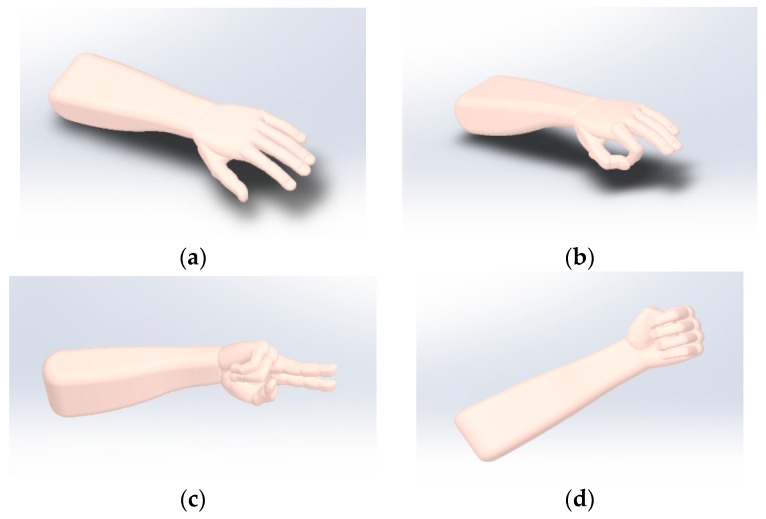
Gesture models: (**a**) stretched hand; (**b**) W-shaped gesture; (**c**) V-shaped gesture; (**d**) fist.

3D modeling software SolidWorks is used to establish different gesture models. All these models are imported into Maxwell, which is an electromagnetic field simulation software [26]. A total of four different gestures are drawn, including stretched hand, W-shaped gesture, V-shaped gesture, and fist, as shown in Figure 3.

**Figure 4:**
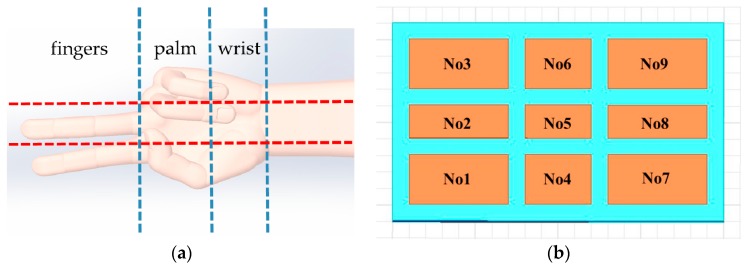
Design of sensing electrodes: (**a**) Division of hand region; (**b**) Arrangement of sensing electrodes

According to the size of the ordinary human hand, the area of the sensing electrode of the detection system is determined to be 20 cm*12 cm, which is slightly larger than that of the hand. The hand can be divided into three areas: fingers, palm, and wrist. The fingers can be divided into three parts to detect whether the fingers of the hand stretch. Correspondingly, the palm is also divided into three parts to detect whether the fingers of the hand bend. The human wrist is very flexible. The wrist is divided into three parts to measure the pitch, yaw, and roll of its, as shown in Figure 4a. Therefore, the detection system is set with nine sensing electrodes, and the sizes of the sensing electrodes are also different according to the proportion of the parts occupying the hand.

Create panel area matrix, as shown in Figure 4b.

(5) $$\left[\begin{array}{ccc} No1 & No2 & No3\\ No4 & No5 & No6\\ No7 & No8 & No9 \end{array}\right]$$

The size of *No*1, *No*3, *No*7, *No*9 area is 6 cm × 3 cm, the size of *No*2, *No*8 area is 6 cm × 2 cm, the size of the *No*4, *No*6 area is 4 cm × 3 cm, and the size of *No*5 area is 4 cm × 2 cm.

After the simulation model is established, the materials, solution domain, excitation source, and boundary conditions of each part of the system are set. The material of the sensing electrode, the emitting electrode, and the system ground is set to be copper, and the insulating material is set to be FR4 (a common material for Printed Circuit Board). The material properties of the human hand are set, including the relative dielectric constant of 77 (slightly less than that of water). The effective detection area of the system is within a sphere centered on it with a radius of 50 cm, so the solution domain is set to be a sphere with a radius of 5 m, and the material of this area is set to be air. The excitation source is set on the emitting electrode, and the voltage amplitudes are 0 V, 0.8 V, 1.5 V, 2.5 V, 3.3 V respectively, and the solution domain boundary and the system ground are set to be 0 V. The simulation calculation is performed to simulate the change in potential on the sensing electrodes under the alternating voltage. The potential changes on the sensing electrodes when the hand is 1 mm, 20 mm, 40 mm, 60 mm, 80 mm, 100 mm away from the detection electrode for the four kinds of gestures are obtained.

**Figure 5:**
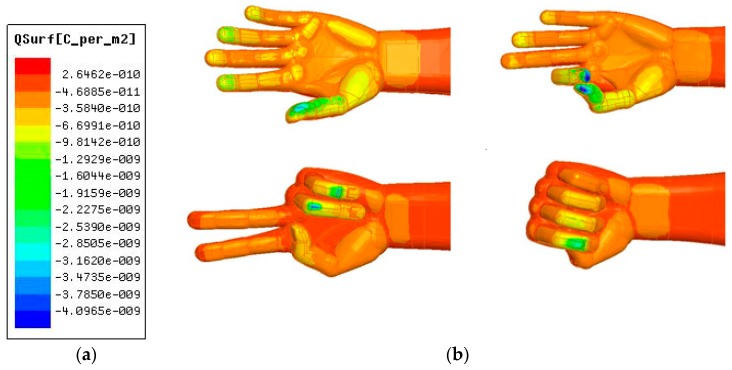
The inductive charge density on the hand: (**a**) Spectral-based charge density meter; (**b**) Charge density distribution of 4 gestures.

Figure 5 shows the inductive charge density on the hand when the 4 gestures are 1 mm away from the sensing electrodes under the emitting electrode voltage of 3.3 V. Since the emitting electrode is loaded with a positive voltage, the hand is negatively charged. The colors in the figure are from red to purple, indicating that the negative charge density gradually increases. The charge density of the blue and purple parts exceeds −3 × 10^−9^ C/m^2^, and the charge density of the yellow and green parts is −6 × 10^−10^ ~ −2.5 × 10^−10^ C/m^2^, the orange and red portion has very little charge, and its effect is basically negligible. Except that the distribution of charge for stretched hand is relatively uniform, for the other three gestures charge is relatively concentrated. Since the difference in distributions of charge for each gesture is obvious, it can be inferred that there is a difference in the potential change on the sensing electrodes.

**Figure 6:**
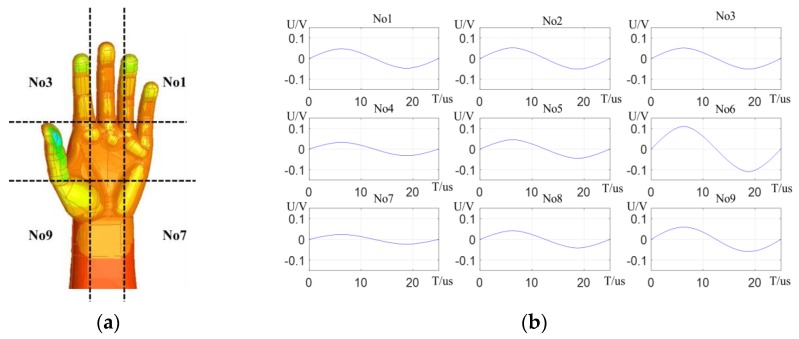
Simulation Analysis of Stretched hand: (**a**) Simulation model of Stretched hand; (**b**) Sensing electrodes potential relative change curves.

Spline interpolation is performed on the discrete sequence composed of the potential values of the sensing electrodes under different emitting electrode voltages. The variation of the sensing electrode potential in one cycle of the alternating excitation source is simulated, and the effects of the four gestures on the potential of the sensing electrodes are analyzed. Figure 6a shows a simulation model diagram of a stretched hand, and Figure 6b shows the relative change curve of the potential for stretched hand. The left thumb is closest to the detection panel, so the overall potential of the sensing electrodes in the palm region changes greatly, and the potential of the electrode in *No*6 region changes the most, exceeding 0.1 V. At the same time, due to the full extension of the fingers, the potential changes of the electrodes in the upper layer are relatively uniform.

Making a W-shaped gesture, the thumb and the index finger are closest to the detection panel, and the hand charge is concentrated here, so the potential change of the *No*6 region electrode is much larger than those of the remaining electrodes, which is almost 0.5 V. The potential of the *No*5 region electrode is relatively large, but is only 0.2 V, and the electrode potential changes in other regions are less than 0.1 V. Making a V-shaped gesture, the curved little finger, ring finger, and thumb are closest to the detection panel, so the potential of the palm and the wrist area changes greatly. The average potential change is more than 0.3 V. When a fist is above the sensing electrodes, charge is concentrated on the curved fingers, the potential change of the electrode in the finger area is extremely small, the potential change of the electrode in the palm area is the largest, and the potential change of the electrode in the *No*6 region exceeds 0.35 V.

When the distance between the hand and the sensing electrodes is 1 mm, the difference in the potentials of all electrodes under different gestures is obvious, and the degree of discrimination is high. However, the magnitude of the potential change is limited to a maximum of 0.5 V. As the distance between the hand and the electrode increases, the amplitude of the potential change further decreases, and the feature extraction difficulty increases. Taking the W-shaped gesture as an example, the maximum potential change of the sensing electrodes is calculated when the distance between the hand and the electrode is 1 mm, 20 mm, 40 mm, 60 mm, 80 mm, and 100 mm, respectively. When the distance reaches 40 mm, the maximum electrode potential change is less than 30 mV, and even less than 10 mV for some electrodes. According to the operator’s habits, the distance between the hand and the electrode is usually within 150 mm. Therefore, the detection system not only needs to improve the structure, but also design the signal-processing circuit to adjust the potential of the sensing electrodes, in order to increase the detection distance of the system, so that it can meet the demand of practical applications. 

**Figure 7:**
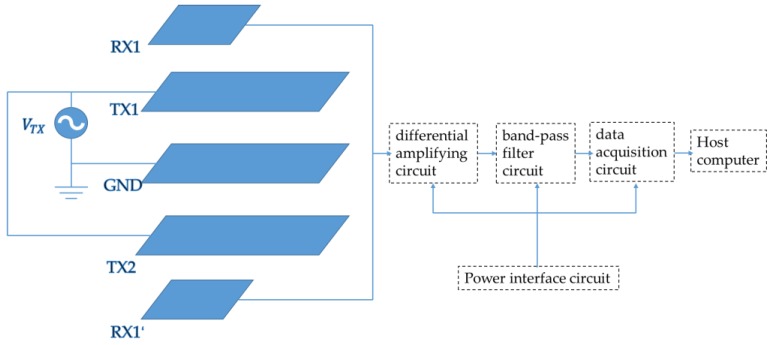
Structure and signal-processing circuit design of the detection system. The signal-processing circuit includes a differential amplifying circuit, a band-pass filter circuit, a data acquisition circuit, and a power interface circuit.

The structure of the detection system is shown in Figure 7. The emitting electrode *TX2* and the sensing electrode *RX1*′ with the same structure as above are designed with the system *GND* as symmetry plane. Due to the shielding effect of *GND*, the upper and lower electrodes do not affect each other so that could be regarded as two independent systems. The excitation source is applied to the two emitting electrodes *TX1* and *TX2*, and the potential values on the sensing electrodes *RX1* and *RX1*′ are basically the same. After the hand enters the electric-field detection range, the potential on the sensing electrode *RX1*′ changes under its effect, and the potential of the sensing electrode *RX1* does not change, so the potential difference between *RX1* and *RX1*′ changes. *φ_RX1_* and *φ_RX1′_* are input to the differential amplifier circuit for filtering. Finally, the voltage signal is transmitted by the data acquisition system to the host computer.

**Figure 8:**
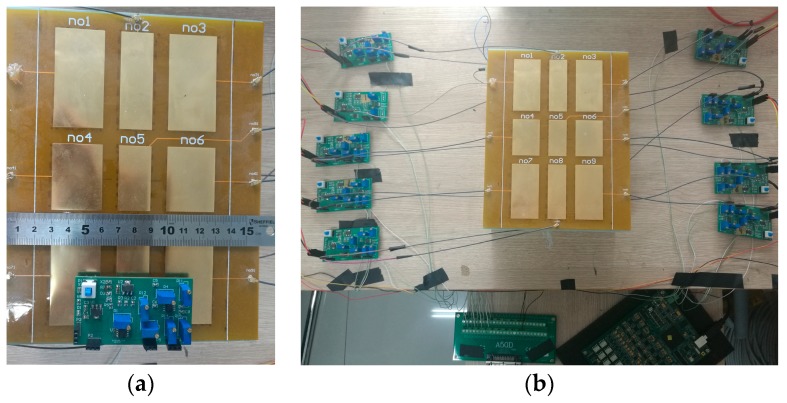
Detection system which includes detection panel, signal-processing board, data acquisition card: (**a**) The size of various parts of the detection system; (**b**) The location of the various parts of the detection system.

The electric-field detection system is shown in Figure 8. The sensing electrode layer is made of a flexible printed-circuit board (FPC), the insulating layer is made of a 10 mm thick epoxy resin plate (FR4), and the emitting electrode and system *GND* are made of brass film. To increase the detection range of the system and reduce the radio wave interference in the space, an alternating signal with amplitude of 5 V and frequency of 40 kHz is applied to the emitting electrode. According to the simulation result, the gain of the differential amplifying circuit is adjusted to be 30. To reduce the effect of 50 Hz power frequency signal in space on the signal, a fourth-order Bessel band-pass filter circuit is designed with a center frequency of 40 kHz and a bandwidth of 1 kHz. The acquisition system selects the USB bus-based data acquisition card, with the range of ± 10 V, and the sampling frequency of 1 MS/s. It mainly consists of the power supply, signal source, oscilloscope, detection electrode, signal-processing circuit, and data acquisition system.

Since the sensing electrodes of the detection system have different sizes, the gain of the signal-processing circuit needs to be fine-tuned to ensure that the sensitivities of all electrodes are the same. The metal ball is suspended above the center of the detection panel, and the height of the ball is adjusted from 10 mm to 150 mm at an interval of 10 mm to collect the potential change of the sensing electrodes at different distances. The gain of the surrounding electrode signal-processing circuit is adjusted with reference to the change in the center sensing electrode potential.

**Figure 9:**
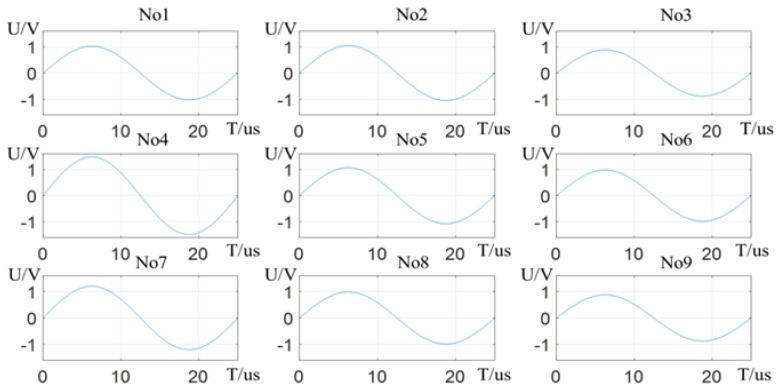
Sensing electrodes potential difference change curve.

The electric-field detection system is tested. The stretched right hand of the operator lifts flat above the detection panel, and the wrist is 80 mm away from the panel. The potential difference curves of the system sensing electrodes are shown in Figure 9. It can be seen that the potential difference of the sensing electrodes in the palm region is relatively large, and the potential of the electrode in the *No*4 region is the largest, which is close to 1.5 V. Since the right thumb is closest to the detection panel, the overall potential difference of the [*No*1, *No*4, *No*7] region electrodes is larger than that of regions [*No*3, *No*6, *No*9]. The distribution of charge on the stretched hand is relatively scattered, so the change in the potential difference of the region electrodes is relatively the same, basically about 1 V.

The measurement and simulation results under the four gestures are compared to know that it has high similarity, which verifies the effectiveness of the detection system and signal-processing circuit. The conditioning of the sensing electrodes potential signal by the signal-processing circuit not only increases the detection distance of the system, but also improves the discrimination of different gesture features.

### 2.3. Gesture-Recognition Method

Gestures are characterized by diversity and difference, and the gestures made by operators are different. The electric-field detection system needs to extract the gesture features from different hand states. We set several common hand states. State 1 is for the hand keeping flat above the electrode, state 2 is for the hand lifting, state 3 is for the hand sagging, state 4 is for the hand side spinning, state 5 is for the hand side shifting. The detection system can recognize 4 gestures in these 5 states: stretched hand, fist, V-shaped gesture, and W-shaped gesture. The distance between the wrist and the panel is taken as the standard distance. After many tests, the comfort distance for operator is 60 mm ~ 90 mm. In this range, the operator can freely change the hand state. This paper mainly studies the characteristics under different hand states and postures under the comfortable distance.

1600 sets of different gesture data of 4 operators in 5 states acquired. We collect 80 sets of data for each gesture of each operator in each state, and randomly select half of them as training set samples. Therefore, 800 sets of data are used to perform parameter training of the recognition algorithm, and the remaining data is used to verify the accuracy of the identification method.

In different hand states, there is a significant difference in the relative distance between the hand and the sensing electrodes of the system so that the potential of each sensing electrode is obviously different. In comparison, different hand postures have less influence on the potential of the sensing electrodes. Therefore, gesture recognition is performed in two steps, first determining the state of the hand, and then distinguishing the gesture.

The detection system divides the space into 9 parts by the sensing electrodes. The potential matrix *V* defining the composition of the sensing electrodes is,

(6) $$V=\left[\begin{array}{ccc} e1 & e2 & e3\\ e4 & e5 & e6\\ e7 & e8 & e9 \end{array}\right]$$

Therefore, the total potential sum of the sensing electrodes is *V_E_* = ∑*e_i_* (*i* = 1, 2, …, 9), the standard deviation of the sensing electrodes is *σ*. The matrix is a basic attribute of a gesture, which could be combined and transformed to form important features of the recognition gesture. First, some elements of the potential matrix are combined to analyze the change of the potential in the partial areas, including a potential matrix row vector [*x*1, *x*2, *x*3], a column vector [*y*1, *y*2, *y*3], upper right region *z*1 composed of *e*2 and *y*3, and lower right region z2 composed of e8 and y1. Calculating the sum of the elements in each area could be expressed as [*V_x_*_1_, *V_x_*_2_, *V_x_*_3_], [*V_y_*_1_, *V_y_*_2_, *V_y_*_3_], [*V_z_*_1_, *V_z_*_2_].

In view of the difference in gestures, even if one person repeats the same gesture, there will be different, so the potential changes of the sensing electrodes are different. However, the proportional relationship of the electrode potentials is determined, so it is normalized.

(7) $$D=\left[\begin{array}{ccc} d1 & d2 & d3\\ d4 & d5 & d6\\ d7 & d8 & d9 \end{array}\right]=\left[\begin{array}{ccc} e1 & e2 & e3\\ e4 & e5 & e6\\ e7 & e8 & e9 \end{array}\right]/\sum_{i=1}^{9}e_{i}$$

The sum of normalized potentials of the regions [*x*1, *x*2, *x*3], [*y*1, *y*2, *y*3] and [*z*1, *z*2] are [*dx*1, *dx*2, *dx*3], [*dy*1, *dy*2, *dy*3] and [*dz*1, *dz*2] respectively. According to the test data, the potential distributions of all regions indifferent hand states are different. *V_E_* decreases significantly when the hand lifts, and increases significantly when the hand drops. When the hand spins to the right, the normalized potential and [*dy*1, *dy*2, *dy*3] increase step by step. When the hand moves to the left, *dz*1 increases and *dz*2 decreases. Through the statistics of the test data, the characteristics of the specific hand state are extracted, as shown in Table 1.

The gesture characteristics under different hand states are different. In general, the stretched hand means stretching all fingers, therefore, the standard deviation σ is relatively small. The fingers of the fist are all bent, so the difference between *dx*1 and *dx*2 is large. Making the V-shaped gesture means that three curved fingers are closer to the electrodes, so *V_E_* is larger. Making the W-shaped gesture means that the index finger and thumb are closer to *y*1, so the difference between *dy*1 and *dy*3 is larger. The remaining features are determined based on different hand states. The hand posture characteristics of each hand state are shown in Table 2.

Based on the hand state characteristics, the decision tree model is constructed using the C4.5 algorithm [27]. First, the five hand states are marked as *x_i_* (*i* = 1, 2, …, 5), and the probability of occurrence in the data set is *p*(*x_i_*). Entropy of data set *S* before splitting could be expressed below.

(8) $$E(S)=-\sum_{i=1}^{5}p(x_{i})log_{2}p(x_{i})$$

Calculate the sum of the entropies of all data sets divided by feature *T*,

(9) $$E_{T}(S)=-\sum_{j=1}^{m}\frac{\left|S_{j}\right|}{\left|S\right|}E_{j}(S)\;(T=1,2,3,4)$$

Where m represents the number of data sets after splitting, and calculates the difference in entropy before and after splitting, i.e., the information gain.

(10) $$InfoGain(S,T)=E(S)-E_{T}(S)\;(T=1,2,3,4)$$

The split information of the data sets divided by feature *T* is,

(11) $$SplitInfo(S,T)=-\sum_{j=1}^{m}\frac{\left|S_{j}\right|}{\left|S\right|}log_{2}\frac{\left|S_{j}\right|}{\left|S\right|}\;(T=1,2,3,4)$$

Information gain rate could be deduced from above equations.

(12) $$InfoGainRation(S,T)=\frac{InfoGain(S,T)}{SplitInfo(S,T)}\;(T=1,2,3,4)$$

The C4.5 algorithm measures each feature by the information gain rate. The greater the information gain rate, the higher the purity of the data set after splitting.

According to the formula above, the information gain rate of each feature of the first splitting point is calculated to be [1, 0.6837, 0.7624, 1], and it can be seen that feature 1 and 4 are the optimal features for the initial splitting. On the whole, the information gain rate of the hand features is generally higher, and the feature differentiation is high.

For the new data set generated after splitting, the information gain rate of different features is calculated again to obtain the optimal splitting feature. Splitting is implemented through constant iterative operations until all features are traversed. After determining the state of the hand, the decision tree model is established according to the hand posture feature to perform gesture recognition.

## 3. Results

The decision tree model is verified using 800 sets of data. The results are shown in Table 3.

Overall, the correct rate of gesture recognition is 91.6%. When the hand lifts, the distance between all parts of the hand and the sensing electrodes increases, and the recognition correct rate is only about 85%. In contrast, when the hand sags, the recognition correct rate increases significantly to 95.8%. This is consistent with the results of the simulation. When the distance between the two is relatively close, the gesture feature discrimination is high, but as the distance increases, the feature extraction difficulty increases, resulting in a decrease in recognition correct rate. Among the four gestures in different states, the recognition rate of the W-shaped gesture is the lowest, which is 83.5%, and the recognition rate of the fist gesture is the highest, reaching 96.5%.

Dynamic gestures can be viewed as continuous hand shapes on a certain time axis. When the hands shape changes, the detection system needs to respond in real time. The excitation source frequency used by the detection system is 40 kHz. To improve the accuracy of recognition, it is usually necessary to collect data of more than 100 cycles, adding the delay time of the circuit, the system gesture-recognition period is about 3 ms. 200 sets of different dynamic gesture data are collected and identified by the decision tree model. The dynamic gestures currently tested consists of two static hand shapes, which include hand states transition under the same gesture and hand gestures transition under the same state. These dynamic gestures can be used to control the direction and trajectory of the robot and the motion of its robotic arm. The results are shown in Table 4.

The result can meet the basic requirements of the gesture-control system, and the recognition correct rate is 87.5%. When the stretched hand in the flat state lifts or sags, the potential of each sensing electrode changes significantly, especially the [*No*1, *No*2, *No*3] sensing electrode, so that the recognition accuracy is relatively high. In comparison, when the hand moves or rotates sideways, the system could correctly recognize the change of state, but may misidentify gestures. The stretched hand remains in flat state and makes a fist, which causes a decrease in the amplitude of the change in the potential of [*No*1, *No*2, *No*3] electrode, and an increase in the amplitude of the change in the potential of [*No*4, *No*5, *No*6], thus having a high recognition accuracy rate.

## 4. Discussion

In the paper, we present a detection system based on low frequency alternating electric field, which can not only accurately identify static gestures in multiple states, but also track the change process of hand postures. Overall, the correct rate of gesture recognition is over 90%, and the dynamic response period is less than 5 ms. 

Our future work will optimize the system structure, and improve the integration of signal-processing circuits so that the portability of the system is improved. In addition, we should extract more gesture features and optimize the algorithm to further improve the recognition accuracy.

The detection system consists only of the detection electrodes and the insulating material, and the structure is relatively simple, so the manufacturing cost is low. Meanwhile, the system detects the postures of the hand through changes in the spatial electric field, so it is less susceptible to the surrounding environment, such as light or sound. In addition, the induced current of the sensing electrode is weak, so the power consumption of the system is low. Therefore, it could keep working efficiently for a long time in complex and harsh outdoor environments. In particular, the sensing electrodes could be covered with any insulating material as a protective layer, regardless of surface structure and reflectivity. Similarly, even if a non-conductive object is located between the hand and the electrode, there are no problems with occlusion and blind spots, which may occur in optical system. We could attach it to the robot as a gesture-control system, and then manipulate it to carry out related work. Furthermore, it could be used for operation control of virtual reality, somatosensory games, and other visualization systems to control avatar actions and scene switching. The significance of our design of the sensor is to adopt a new gesture-detection system, which has unique advantages in some respects, and provides more ways and methods for the efficient implementation of human-robot interaction in different scenarios. 

## Figures and Tables

**Table 1:** Hand state characteristics.

Features
*dy*1 < *dy*2 < *dy*3
*dz*1 > 0.6 && *dz*1 − *dz*2 > 0.3
*V_E_* > 11, 6.5 < *V_E_* <11, *V_E_* < 6.5 Max [*V_x_*_1_, *V_x_*_2_, *V_x_*_3_] > 5

**Table 2:** Hand posture characteristics.

States	Features
Flat	*V_E_* < 7.2
	*σ* < 0.48
	*dx*2 − *dx*1 > 0.12
Lifting	*dy*1 − *dy*3 > 0.13, 0.08 < *dy*1 − *dy*3 < 0.13, *dy*1 − *dy*3 < 0.08
	*dx*3 − *dx*2 > − 0.015
Drooping	*V_E_* < 11.5
	*σ* > 0.65
	*dx*1 < 0.36
Side spin	*V_E_* > 7.5
	*dx*2 − *dx*1 < 0.11
	*dy*3 − *dy*1 < 0.085
Side shift	*σ* < 0.45
	*dx*2 − *dx*1 < 0.65
	*d*7 − *d*5 < 0.1

**Table 3:** Static gesture verification.

Gestures	Verification		
	Correct	Incorrect	Correct Rate
Flat (stretched)	39	1	97.5%
Flat (fist)	33	7	82.5%
Flat (V-shaped)	40	0	100%
Flat (W-shaped)	40	0	100%
Lifting (stretched)	34	6	85%
Lifting (fist)	40	0	100%
Lifting (V-shaped)	28	12	70%
Lifting (W-shaped)	35	5	87.5%
Sagging (stretched)	40	0	100%
Sagging (fist)	40	0	100%
Sagging (V-shaped)	40	0	100%
Sagging (W-shaped)	35	5	87.5%
Side spinning (stretched)	32	8	80%
Side spinning (fist)	40	0	100%
Side spinning (V-shaped)	40	0	100%
Side spinning (W-shaped)	27	13	67.5%
Side shifting (stretched)	40	0	100%
Side shifting (fist)	40	0	100%
Side shifting (V-shaped)	40	0	100%
Side shifting (W-shaped)	30	10	75%

**Table 4:** Dynamic gesture verification.

Dynamic Gestures	Verification		
	Correct	Incorrect	Correct Rate
Flat (stretched)→Side shifting (stretched)	34	6	85%
Flat (stretched)→Side spinning (stretched)	31	9	77.5%
Flat (stretched)→Lifting up (stretched)	39	1	97.5%
Flat (stretched)→Sagging (stretched)	35	5	87.5%
Flat (stretched)→Flat (fist)	36	4	90%
